# Job loss and job instability during the COVID-19 pandemic and the risk of depression and anxiety among Swedish employees

**DOI:** 10.1016/j.ssmph.2023.101424

**Published:** 2023-05-04

**Authors:** Sandra Blomqvist, Robin S. Högnäs, Marianna Virtanen, Anthony D. LaMontagne, Linda L. Magnusson Hanson

**Affiliations:** a Stress Research Institute at Department of Psychology, Stockholm University, Stockholm, Sweden; b School of Educational Sciences and Psychology, University of Eastern Finland, Joensuu, Finland; c Institute for Health Transformation, and School of Health & Social Development, Deakin University, Geelong, VIC, Australia

**Keywords:** COVID-19, Unemployment, Furlough, Downsizing, Depression, Anxiety

## Abstract

The COVID-19 pandemic led to permanent and temporary job losses but the mental health consequences of different types of employment transitions are not well-understood. In particular, knowledge is scarce concerning furloughs, which was a common job protection strategy in many high- and upper middle-income countries during this crisis. This study focuses on how different types of job instability and job loss during the pandemic influences depression and anxiety in the context of Sweden.

A subset of participants from the Swedish Longitudinal Occupational Survey of Health were contacted in February 2021 and again in February 2022. A total of 1558 individuals participated in either or both waves and worked before the pandemic. We examined whether i) workplace downsizing, ii) furlough, or iii) unemployment/job loss were associated with depression and anxiety over this one-year period during the pandemic. Logistic regression models with cluster-robust standard errors were estimated, adjusting for sociodemographic factors and prior mental health problems. Effect modification by sex and prior mental health problems was also examined.

In comparison to stable employment, being furloughed was unrelated to mental health, while experiencing workplace downsizing during the pandemic was associated with an increased risk of anxiety (adjusted Odds Ratio (OR) = 2.09, 95% Confidence interval (CI) = 1.08–4.05). Job loss/unemployment increased the risk of depression (OR = 1.91, 95% CI = 1.02–3.57) compared to being stably employed, but the risk estimate crossed unity when considering prior mental health status. No effect modification by sex or by prior mental health problems was found.

This study found that while job loss and downsizing during the COVID-19 pandemic were associated with depression and anxiety, respectively, being furloughed was not. These findings thus suggest that job retention schemes in the form of short-time work allowances, as implemented in Sweden during the COVID-19 pandemic, may prevent mental health problems among employees during economic crises.

## Highlights

• Job loss and health during recessions has been studied before but less is known about furloughs.
• We used a nationally representative sample of the Swedish working population.
• Furlough was unrelated to anxiety and depression compared to stable work situations.
• Job loss was associated with depression and workplace downsizings with anxiety.

## 1 Introduction

The COVID-19 pandemic affected employees and their employment situations substantially. In order to mitigate the spread of the SARS-CoV-2 virus, restrictions were introduced that affected our social lives and geographical mobility were introduced, with consequences for businesses, organizations, and employees. Due to a decreased demand for services and supply chain disruptions, many employers were forced to cut employee costs. This is reflected in unemployment rates which rose by 2% to a level of 9.2% in Sweden during the first couple of months of the pandemic, which is comparable to the rates observed during the Great recession in 2008 (Statistics Sweden, 2022). Approximately 2% of the Swedish working population were notified of layoff between March and June 2020, with large variability across industries (Swedish Public Employment Service, 2021). In the hotel, restaurant and cultural service sectors for instance, nearly 8% of the employees were notified of layoff. To support work during the pandemic and preserve employer-employee links, Sweden, along with many other countries, either implemented or supported pre-existing short-time work allowance schemes. This meant that many employees, instead of being dismissed, were put on furlough and kept their employment contracts, but worked less with a reduced and partly state financed salary. In Sweden there was a pre-existing system of support for short-time work which could be activated by the government if; 1) a considerable steep economic downturn is present or soon awaiting, or 2) that an implementation of the support would not impair an otherwise needed structural change of the labor market. The system was implemented in 2014 as a response to the lack of such a scheme during the financial crisis in 2008, but it has never been activated, including during the COVID-19. Instead, a legislative system that was still in preparation, which was designed to complement the above-mentioned system and to replace local agreements on short-time work, was launched prematurely and tailored to the context of the pandemic (Swedish Government Official Reports SOU, 2018; Swedish Government Official Reports SOU, 2022). The state was made responsible for 75% costs related to the short time work and the general rule of excluding public sector employers was somewhat eased up. In addition, pre-existing levels of reduced work hours (20%, 40% and 60%) were extended to the possibility of reducing hours up to 80%, alongside more generous replacement levels (up to a wage cap of ∼4000 EUR).

All individuals who were employed three months prior to the short time work allowance decision were eligible for support. However, support eligibility requirements for employers included a priori attempts to cut their costs, which included layoffs. This primarily affected employees on temporary contracts, which are more often held by women, young people and migrants, and specifically within the service and retail industries (Statistics Sweden, 2020), which were particularly hit hard by the pandemic. Overall, 20% of the Swedish employees were furloughed at some point during the pandemic (Swedish Agency for Economic and Regional Growth, 2021), and the scheme likely averted some unemployment and widening of income inequalities in Sweden during the pandemic (Angelov & Waldenström, 2023; Swedish Agency for Growth Policy Analysis, 2022). According to the OECD (OECD, 2021), these types of schemes also likely limited the mental health impacts of the pandemic on workers. A difference with the Swedish scheme and those in other Nordic countries and the UK, was the possibility of only reducing work hours to a maximum of 80%, whereas in other countries work hours could be reduced by 100%. Otherwise, replacement rates and salary caps were comparable, with the exception of Norway who provided full reimbursement the first 20 days (Juranek, Paetzold, Winner, & Zoutman, 2021). Short-time work allowance schemes previously have not been as widely used in Sweden (on a national basis) nor in many other countries (Ebbinghaus & Lehner, 2022), and thus, the consequences of them merit further attention.

Although the COVID-19 restrictions may have had both negative and positive effects on mental health, a general increase in mental health problems during the pandemic has been repeatedly reported in the general population (Santomauro et al., 2021; Wu et al., 2021). Occupation and work environment seem to be important, and consistent with expectations, research has suggested that an increase in job loss and job insecurity across the pandemic negatively affected mental health (Posel et al., 2021; Shoss, 2021; Wilson et al., 2020). Some earlier findings have proposed that mental health problems in terms of emotional exhaustion are more marked for an actual job loss than the anticipation of job loss (Halbesleben et al., 2013). Some scholars thus argue that different employment statuses need to be examined separately (Halbesleben et al., 2013; Mohr, 2000). On the other hand, antecedents, moderators and consequences, appear to be similar for job insecurity, anticipation of job loss and actual job loss (De Witte et al., 2019; Dooley, 2003). Some scholars argue that the shared concern is ultimately about job loss, and therefore the concepts capture different stages of the same underlying process and should be assessed as a continuum (De Witte et al., 2019; Dooley, 2003).

Theoretically, associations between job loss, job insecurity and subsequent mental health consequences have been interpreted in the light of the conservation of resource (COR) model on stress, which proposes that psychological stress reactions are elicited when an individual's retention, protection or building of highly valued resources is threatened (Hobfoll, 1989). In the context of employment as a resource, a more detailed description of what exactly it is that employments contain that a person may want to protect, is offered by the latent deprivation theory (Jahoda, 1982). The theory, which is often used in combination with the COR model (Selenko & Batinic, 2013; Vander Elst, Näswall, Bernhard-Oettel, De Witte, & Sverke, 2016), stipulate that employments includes a set of manifest and latent functions that are beneficial to our mental wellbeing; however when a job is lost or threatened, so are these functions, which increases the risk of mental distress. Jahoda argues that income is a manifest function, whereas time structure, activity, collective purpose, status and social contacts are latent functions.

According to a recent meta-analysis, both sets of functions are associated with mental health and employed versus unemployed people generally have better access to them (Paul, Scholl, Moser, Zechmann, & Batinic, 2023), particularly with respect to status, income and time structure (Paul et al., 2023). Applications of the model to the association between job insecurity and mental health has led to mixed result in terms of whether manifest (Selenko & Batinic, 2013) or latent (Vander Elst et al., 2016) have more explanatory power.

Other theoretical models such as the transactional stress theory, posits that prolonged worry about a potential job loss can be just as or even more stressful than an actual job loss due the difficulty of coping with the undefined (Lazarus & Folkman, 1984). Empirically, studies investigating job instability and job disruptions during the COVID-19 pandemic, have found that experiences of job loss and furloughs, compared to stable employment, were associated with more depressive symptoms and psychological distress (Abrams et al., 2022; Escudero-Castillo et al., 2021), although one study did not find an association with anxiety (Abrams et al., 2022). Some studies have also shown stronger associations between unemployment and job loss and mental health than being furloughed (Abrams et al., 2022; Wang et al., 2022; Wels et al., 2022). Yet others have shown that furlough was unrelated to psychological distress while being permanently laid off increased the risk thereof, compared to employees with no experience of employer cuts (Burchell, Wang, & Kamerāde, 2020; Ferry, Bunting, & Rosato, 2021). According to a South African study, fewer depressive symptoms was observed among employees on paid furlough whereas no positive effects were seen among those on unpaid furlough, compared to employees who lost their job (Posel et al., 2021). A study from the UK found no difference in mental distress between paid (furlough) or unpaid (reduced working hours) temporary leave (Wang et al., 2022). Compared to leaving or losing one's job, a temporary leave, paid or unpaid, was only associated with better mental health among men (Wang et al., 2022). Finally, in a study on Finnish public sector employees, those who experienced team reorganizations during the pandemic had similar decreases in psychological distress as employees who did not, while being assigned new work tasks seemed to increase the risk of psychological distress (Ervasti et al., 2021). Taken together, results from studies investigating job instability during the COVID-19 pandemic have been mixed and more studies are needed. in particular with respect to furloughs, by which potential consequences seems to depend on individual contexts, including financial circumstances (Matthews, 2021). Our aim was to extend the literature by investigating the association between job instability during the COVID-19 pandemic and the risk of mental health problems in the Swedish context. Specifically, we compared experiences of job loss, furlough and workplace downsizing to those in stable employment situations, with regard to anxiety and depression.

## 2 Material and methods

**Fig. 1:**
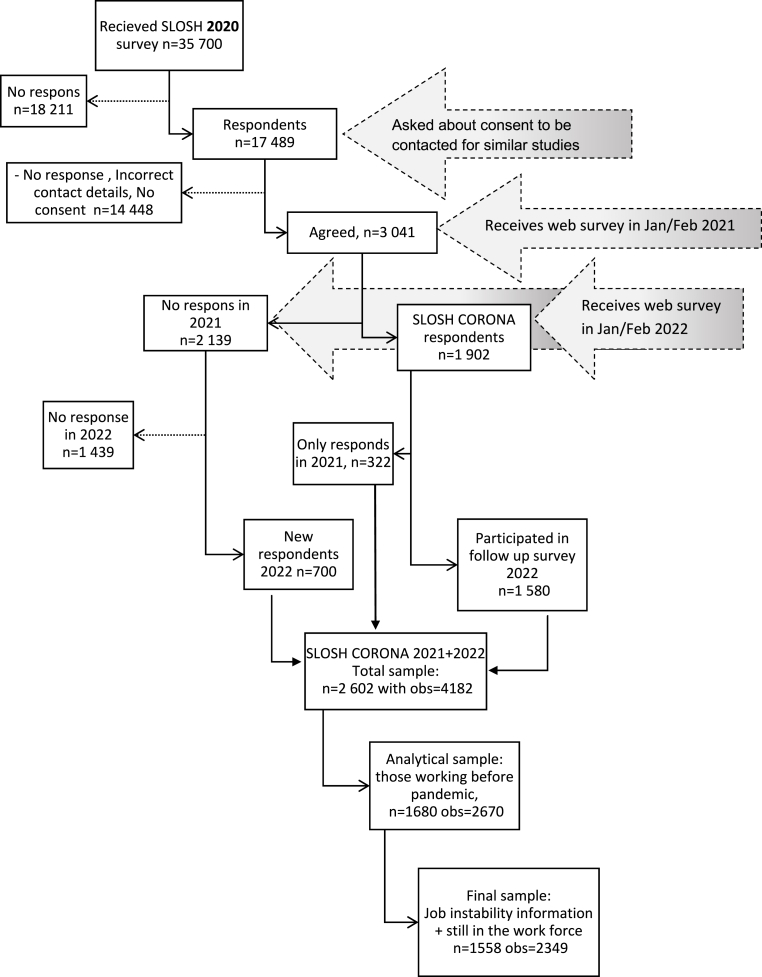
Selection of study subjects.

The study relies on information from a sub-sample of individuals participating in the Swedish Longitudinal Occupational Survey of Health (SLOSH) which collects data on work, private life and health on a sample of working age Swedes (n ~ 41 000). The SLOSH cohort consists of individuals participating the Swedish Labor Force Survey between 2003 and 2011 and, who at that time point, reported that they were in employment and took part in the Swedish Work Environment Study. The ethics committee of Stockholm, Sweden, has approved the SLOSH project and participants are informed by the voluntariness in participating. A thorough description of SLOSH is available as a published article (Magnusson Hanson et al., 2018). Auxiliary to the regular bi-annual SLOSH data collection, the “SLOSH Corona” collected similar information in the context of the COVID-19 pandemic. A link to the SLOSH Corona survey was emailed to participants who responded to the SLOSH 2020 survey and agreed to be subsequently contacted and who provided a correct email address and agreed to receive an invitation to a web-based survey (n = 3 041, 17%). In total n = 1902 (63%) responded to the SLOSH Corona web survey in Jan–Feb 2021.The majority (n = 1580) responded to the follow-up survey one year later, while n = 322 dropped out, resulting in an attrition rate of 18%. When comparing those only responding to the first wave to those responding to both waves, we found no significant differences in baseline characteristics (i.e., sex, age, educational degree, occupational class, civil status, mental health problems in 2018, nor self-reported anxiety or depression at Wave 1) in chi-square and two-sample t-tests. However, those who only responded in Wave 1 included a higher proportion of people in paid work prior to the pandemic than those responding to both waves (see Supplementary Table 1 for details). Compared to the general SLOSH cohort, the SLOSH corona sample have slightly higher levels of education and income, but are otherwise similar with regard to mental health, perceived job insecurity and other sociodemographic factors (Blomqvist, Virtanen, Westerlund, & Magnusson Hanson, 2023). At the time of the follow up in 2022, additionally n = 700 individuals were recruited and responded to SLOSH Corona for their first time. In total n = 2602 (86% of those n = 3401 that were eligible for the study) responded to one or both surveys, contributing to 4182 observations. For this study we included only those who worked prior to the COVID-19 pandemic (n = 1680), who remained in the working population, (i.e., eligible for work) and provided information about the key job instability measures and mental health. This yielded an analytical sample of n = 1558 and a total of 2349 observations, (an overview of the sampling steps is available in Fig. 1).

### 2.1 Job instability

Exposure to job instability was determined across two different study periods; from March 2020 (start of the pandemic) to February 2021 and from February 2021 to February 2022. Along a continuum, job instability included the following categories: stable worķ downsizing (without job loss), furloughed (forced reduction in working time and pay without loss of employment) and job loss (becoming unemployed or dismissed). If participants experienced several events, they were coded into the more unstable category along this continuum with job loss as the decisive event. This resulted in a variable with mutually exclusive, ordered categories during each period, where those who did not experience any of these conditions, but worked prior to the pandemic and were still in the workforce, as the reference group. Items and responses are described in detail below.

#### 2.1.1 Downsizing

Exposure to downsizing was a composite variable based on answering affirmatively to at least one of the following questions; *Since the start of the COVID- 19 pandemic:* “*Have you received a notification about a potential lay off?”, “Did the company where you were employed declare bankruptcy?”* or *“Have there been organizational downsizings with staff reductions at your workplace?”* Two variables, covering the first and second study period, were created based on the participant's experiences to any of these events (Yes = 1, No = 0). Individuals who only participated in Wave I provided information for the first period, while individuals answering to both surveys or who were recruited into the study in 2022 provided information across the entire timespan. However, for those joining the study in 2022, we only used their information from the second period of the pandemic as we did not have information about their mental health during the first period. Respondents who reported exposure to downsizing but who did not also report furlough or unemployment/job loss during the same period were classified as ‘exposed to downsizing'.

#### 2.1.2 Furlough

In Sweden, state-subsidized short-time work allowance schemes were used during the pandemic. Working time could be reduced to a maximum of 80%, which meant a pay cut of 12% for the employee. Study participants who experienced any degree of furlough, but did not also become unemployed or dismissed during the same period, were classified as exposed to “furlough” during that respective study period.

#### 2.1.3 Job loss

Two dichotomized variables on unemployment or dismissal, classified as “job loss”, were created based on answers “Yes” or “No” to the questions: “Did you become unemployed (or dismissed) during ….” i) the spring or autumn 2020, or ii) the spring or autumn in 2021. Respondents reporting having been dismissed or unemployed were defined as exposed to “job loss” in that respective period.

### 2.2 Mental health

The Generalized Anxiety Disorder 7 item scale (GAD7) with a cut-off ≥10 was used to classify probable cases of generalized anxiety disorders in both study periods (Spitzer, Kroenke, Williams, & Lowe, 2006). Correspondingly, we used the Patient Health Questionnaire 9 items (PHQ9) with a cut-off ≥10 to classify the respondents according to probable caseness of depressive disorders (Kroenke et al., 2001). Both scales ask the respondents about their mood and feelings in the past 2 weeks and have shown high reliability, good diagnostic criterion validity and construct validity (Kroenke et al., 2001; Spitzer et al., 2006). We further created one dichotomous variable collapsing both measures, with 1 denoting depression and/or anxiety and 0 denoting neither depression nor anxiety. This outcome was used primarily with the intent of increasing power in sub analyses.

To account for the presence of anxiety symptoms prior to the pandemic, we used information from the SCL-ANX4, a sub scale to the Symptom Checklist-25, available in the 2018 SLOSH data collection. Sum scores ranged between 0 and 16 and possible cases with anxiety prior to the pandemic were defined based on scores of ≥6 (Søgaard, 2009). Similarly, we defined possible cases with depressive symptoms prior to the pandemic as those with scores above 16 on a sum scale (0–24) from the SCL-CD6, also from SLOSH 2018 (Magnusson Hanson et al., 2014).

### 2.3 Covariates

Based on previous research (Leung CMC, Ho MK, Bharwani AA, Cogo-Moreira H, Wang Y, Chow MSC, Fan X, Galea S, Leung GM, Ni MY., 2022; Xiong et al., 2020), a set of covariates, either related to the exposures of job instability, mental health or both, were accounted for in the statistical models. These included the participants’ age, sex, education (compulsory, upper secondary or university level education), country of birth (Sweden or outside of Sweden) and socioeconomic classification (SEI) of manual or non-manual occupations, all drawn from register-based information from Statistics Sweden. We also included information on civil status (married/cohabiting or single) from SLOSH.

### 2.4 Statistical analysis

Descriptive statistics were presented jointly for all study participants, and separately by job instability indicators (i.e., downsizing, furlough and job loss) and participants in stable work situations. Participants responding to survey 1 and 2 but reporting several job instability experiences across the study period (e.g., downsizing in 2020 and job loss in 2021), are represented in all the corresponding job instability columns in Table 1, in order to illustrate the prevalence of each and every type of exposure. Furthermore, those in stable work situations (denoted stably employed) in Table 1 consists of participants that did not report any of the aforementioned job instability indicators, in any of the surveys.

**Table 1:** Sample characteristics within the total sample and by exposure to job instability at any point in time during the pandemic. Column percent are presented for comparability between groups.

	All n = 1558		Stable work situation		Downsizing		Furloughed		**Job loss**	
			n = 1171 (75%)		n = 173 (11%)		n = 140 (9%)		**n = 98 (6%)**	
	N/mean	%/sd	N/mean	%/sd	N/mean	%/sd	N/mean	%/sd	N/mean	**%/sd**
Health during the pandemic:										
Depression (PHQ9)	135	9	94	8	16	10	13	10	12	**13**
Anxiety (GAD7)	75	5	49	4	13	8	5	4	5	**5**
Women	930	60	728	62	97	56	63	45	56	**57**
Age	55	11	55	11	55	11	53	11	57	**11**
Married/cohabiting	1202	78	913	79	131	76	105	75	71	**73**
Compulsory education	47	3	35	3	5	3	5	4	2	**2**
Upper secondary	491	32	362	31	63	36	47	34	29	**30**
University	1017	65	771	66	105	61	88	63	67	**68**
Manual employee	259	17	183	16	34	20	28	20	20	**21**
Non-manual employee	1278	83	974	84	136	80	109	80	77	**79**
Born in Sweden	1475	95	1117	95	159	92	130	93	90	**92**
Pre-pandemic health:										
Depression	53	4	38	4	8	5	7	6	3	**4**
Anxiety	191	14	142	14	26	18	18	15	11	**14**

We estimated logistic regression models with Huber White robust standard errors to adjust for multiple observations for individuals over time with 95% Confidence Intervals (95% CI) around odds ratio estimates (OR). In the main analyses we present 1) crude models, 2) models adjusting for sociodemographic factors and 3) models additionally accounting for prior mental health problems, separately for anxiety (GAD7) and depression (PHQ9). The main analysis relies on categorization of job instability during the first period of the pandemic for those only partaking in the first survey, job instability status during the second period for those partaking only in the second survey, while statuses during both periods were used for those partaking in both surveys. This ensured comparable time spans from exposure to outcome across all three respondent groups. As mentioned previously, information on exposure for those only partaking in the second wave was also available during the first part of the pandemic but we only had access to information about their mental health information for the late pandemic. Therefore, we chose to restrict their study period to the second period of the pandemic in the main analysis. As a sensitivity analysis though we regressed exposure to job loss/instability using information about their severest experience during the entire study period (i.e., period 1 and 2), on their depression and anxiety reports in the second period (i.e., when their mental health data was available).

We also ran stratified analyses for by sex, and whether or not respondents had prior mental health problems according to SLOSH 2018. We evaluated the presence of effect modification with likelihood ratio tests on the 0.05 significance level, comparing models including and excluding a product term between exposure and effect modifier.

All analyses were performed using SAS 9.4, and proc surveylogistic with specified cluster id were used in the multivariate analysis to account for intra-individual dependence. No sampling weights were included in the statistical models.

## 3 Results

The majority (n = 1 171, 75%), of all the study subjects (n = 1558) had a stable employment situation during the COVID-19 pandemic. Sociodemographic factors and health among the stably employed resembled that of the total study sample, see Table 1. With regard to experienced job instability during the pandemic; downsizing n = 173 (11%) was most common, followed by furlough (9%) and job loss (6%). Apart from the larger proportion of men among employees being furloughed, the furloughed and the downsizing group largely resembled the stably employed with the exception of a larger proportion of younger employees and manual workers in those with unstable employment. The unemployed on the other hand, were older, with lower income, less likely to be married or cohabiting and had the largest proportion of manual employees 21%. A slightly higher proportion of mental health problems were found among all job instability groups compared to the stably employed, (Table 1). Depression was most prevalent among the unemployed (n = 12, 13%) while the prevalence of probable generalized anxiety disorder (n = 13, 8%) was highest for those experiencing downsizing.

### 3.1 Job instability and mental health during the COVID-19 pandemic

Compared to remaining stably employed during the pandemic, downsizing was associated with anxiety (Table 2. Crude OR 2.04, 95% CI 1.14–3.66). The findings remained statistically significant after adjusting for sociodemographic factors (OR 2.09, 95% CI 1.15–3.81), and prior mental health problems (OR 2.09, 95% CI 1.08–4.05).

**Table 2:** Logistic regression models, with robust standard error, on associations between job instability and anxiety by GAD7, crude and adjusted models.

	Crude			Socio-demographics			Prior mental health		
	OR	95%CI		OR	95%CI		OR	95%CI	
Job Instability (ref. = stably employed)									
Downsizing	**2.04**	**1.14**	**3.66**	**2.09**	**1.15**	**3.81**	**2.09**	**1.08**	**4.05**
Furloughed	0.73	0.29	1.84	0.72	0.28	1.85	0.66	0.23	1.91
Job loss	1.42	0.53	3.80	1.45	0.56	3.76	1.18	0.36	3.83
Prior mental health problems (ref. = no)							**3.65**	**2.22**	**5.99**
Male (ref. = female)				0.94	0.61	1.47	1.00	0.62	1.61
Education (ref. = high)									
Low				0.41	0.05	3.15	0.42	0.05	3.58
Medium				1.30	0.81	2.09	1.28	0.77	2.14
Occupation (ref. = non-manual)									
Manual				1.60	0.94	2.72	1.39	0.76	2.53
Age (years)				0.98	0.96	1.00	0.98	0.96	1.01
Married/cohabiting (ref. = yes)				1.25	0.77	2.00	0.93	0.55	1.57
Born in Sweden (ref. = born abroad)				1.39	0.51	3.81	2.03	0.66	6.23

While furlough appeared to be unrelated to both anxiety and depression, job loss was borderline significantly associated with an increased risk of depression (Table 3). After controlling for sociodemographic factors, experienced job loss during the pandemic was associated with depression (OR 1.91, 95% CI 1.02–3.57). However, when including prior mental health problems in the model, the association was weakened and the confidence interval crossed unity.

**Table 3:** Logistic regression models, with robust standard error, on associations between job instability and depression by PHQ9, crude and adjusted models.

	Crude			Socio-demographics			Prior mental health		
	OR	95%CI		OR	95%CI		OR	95%CI	
Job Instability (ref. = stably employed)									
Downsizing	1.22	0.70	2.13	1.26	0.72	2.21	1.20	0.67	2.17
Furloughed	1.17	0.67	2.07	1.18	0.65	2.14	1.15	0.60	2.19
Job loss	1.77	0.95	3.29	**1.91**	**1.02**	**3.57**	1.63	0.77	3.45
Prior mental health problems (ref. = no)							**3.47**	**2.34**	**5.17**
Male (ref. = female)				0.90	0.62	1.30	0.90	0.61	1.33
Education (ref. = high)									
Low				0.48	0.11	2.08	0.50	0.11	2.29
Medium				1.46	0.98	2.18	1.50	0.98	2.32
Occupation (ref. non-manual)									
Manual				1.09	0.67	1.78	1.10	0.65	1.86
Age (years)				0.98	0.96	1.00	0.98	0.96	1.00
Married/cohabiting (ref. = yes)				**1.50**	**1.02**	**2.23**	1.19	0.78	1.81
Born in Sweden (ref. = born abroad)				1.26	0.55	2.87	1.89	0.84	4.27

### 3.2 Effect modification by sex and prior mental health problems

In Table 4, the analyses are stratified by reported symptoms of anxiety or depression in 2018. Estimates suggested similar directions of associations for those exposed to job instability during the pandemic, with or without prior mental health problems. Furthermore, effect modification by prior mental health was not supported according to likelihood ratio test, neither for anxiety nor depression.

**Table 4:** Logistic regression models with stratification by history of depression or anxiety, all models are adjusted for sociodemographic factors.

	Prior mental health problems			No prior mental health problems		
	OR	95%CI		OR	95%CI	
Anxiety (GAD7)						
Job Instability (ref. = stably employed)						
Downsizing	2.03	0.74	5.57	2.09	0.86	5.05
Furloughed	0.45	0.05	4.40	0.82	0.25	2.76
Job loss	1.15	0.20	6.71	1.07	0.26	4.41
**Depression (PHQ9)**						
Job Instability (ref. = stably employed)						
Downsizing	1.43	0.55	3.71	1.07	0.46	2.49
Furloughed	1.07	0.30	3.81	1.27	0.60	2.70
Job loss	0.97	0.27	3.46	1.97	0.86	4.53

*model comparison with and without interaction between exposure and moderator, with likelihood ratio test 0.05 significance level.

Similarly, no statistical support for effect modification by sex was found, although we could see that women experiencing downsizing had a significantly higher risk of anxiety compared to stably employed women, whereas the corresponding OR for men was close to unity (Table 5).

**Table 5:** Logistic regression models with stratification by sex, all models are adjusted for sociodemographic factors.

	Women			Men		
	OR	95%CI		OR	95%CI	
**Anxiety (GAD7)**						
Job Instability (ref. = stably employed)						
Downsizing	**3.13**	**1.57**	**6.23**	1.19	0.37	3.78
Furloughed	0.63	0.14	2.81	0.79	0.23	2.71
Job loss	1.71	0.59	4.91	1.16	0.17	7.84
**Depression (PHQ9)**						
Job Instability (ref. = stably employed)						
Downsizing	1.49	0.78	2.86	1.09	0.40	2.96
Furloughed	1.10	0.46	2.59	1.24	0.55	2.83
Job loss	1.77	0.78	4.00	2.06	0.77	5.53

### 3.3 Sensitivity analyses

As a sensitivity analyses, we also defined exposure to job instability for the group recruited to the survey in 2022 according to job instability/loss as reported across the entire pandemic, not only according to the second period. This meant that the time lag between exposure and outcome for this group could be longer. The increased risk estimate of depression for those with job loss remained in this analysis, but also crossed unity when including prior mental health status. The association between downsizing and anxiety became weaker and no longer statistically significant (data not shown).

## 4 Discussion

This study found that, in a Swedish context, job loss during the pandemic was associated with depression, workplace downsizings with anxiety, but being furloughed was unrelated to either of these adverse mental health outcomes, all compared to remaining in stable work during the pandemic.

In the light of the conservation of resource (COR) theory, which is often used as one explanatory theory in relevant studies, the absence of negative mental health consequences when being furloughed is somewhat counterintuitive. The COR theory stipulate that losing important resources causes strain which eventually can lead to ill-health (Hobfoll, 1989). Halbesleben (2013) have further argued for, and found empirically, an extension to the theory in which he suggests that an actual loss (being furloughed) is more stressful than a threat of loss (threat of furlough) as the resource loss become more salient then (Halbesleben et al., 2013). When being furloughed the employee loses several resources, matching Jahoda's idea of latent and manifest functions of work (Jahoda, 1982), such as parts of their income, social contacts at work, time structure, sense of purpose or belongingness. However, our findings on furloughs, which corroborate previous findings on the COVID-19 pandemic, job instability and mental health, suggest that furlough, is not, or is only limitedly, related to poor mental health (Abrams et al., 2022; Burchell, Wang, & Kamerāde, 2020; Ferry, Bunting, & Rosato, 2021; Wang et al., 2022; Wels et al., 2022).

Inconsistent with Halbesleben (2013), we also find that exposure to downsizing, a work situation that encompasses threats of job loss and heightened job insecurity at the workplace ((Halbesleben et al., 2013)Kivimäki, Vahtera, Pentti, & Ferrie, 2000; Paulsen et al., 2005), increases the risk of anxiety. This finding resonates better with a common interpretation that insecurity about what the future holds, can in many cases, be worse than knowing even when the information about the future is undesirable (Lazarus & Folkman, 1984; Sverke et al., 2002). Further consistent with prior research during the pandemic (Abrams et al., 2022; Burchell et al., 2020; Ferry et al., 2021; Wels et al., 2022), we found that job loss and unemployment increased the risk of depression. This could be explained by a permanent job loss better capturing harmful losses of resources according to the COR theory, whereas partial and temporary detachment from work while keeping the lion's share of one's salary still offers a sense of having access to the resources.

It is further possible that being furloughed may offer a break from work and free time for recovery. A previous study showed that engaging in recovery during furlough reduced the risk of negative consequences such as emotional exhaustion (Halbesleben et al., 2013). However, on average positive lifestyle changes in the Swedish population during the COVID-19 pandemic appear to have been fairly modest (Blom et al., 2021; Elvén et al., 2022). A more likely explanation could be that the job contract is not lost, as opposed to when becoming unemployed, and that the salary was only partially affected. The latter is consistent with the findings of the South African study mentioned earlier, in which those on unpaid leave had a higher risk of depression compared to the actively working employees while those on paid leave did not differ from the actively working group (Posel et al., 2021). The diverging findings on furlough and mental health could partially be attributed to national variations in types of furlough or short time work allowance schemes. In the United States and Spain where negative mental health consequences from furlough have been observed (Abrams et al., 2022), job retention schemes seem to have had a lower uptake and lower replacement rates, compared to in the United Kingdom (Burchell et al., 2020; Ferry et al., 2021; Wels et al., 2022) and in Sweden where more generous schemes were implemented and furlough spells seems unrelated to mental health (Ebbinghaus & Lehner, 2022; OECD, 2020). Being put on furlough could also be interpreted by the employees as a shift from threats of job loss towards a solution to the current lack of demand for their activity in the workplace, and thus replacing the uncertain by the known.

Being on furlough and not at work may also have been associated with a reduced risk of contracting a COVID-19 infection and fewer worries about being infected, both of which have been associated with increased mental health problems during the pandemic, particularly among employees unable to work from home (Alimoradi, Ohayon, & Griffiths, 2022; Liu et al., 2021).

In contrast to our results though, a study on public sector employees in Finland experiencing organizational changes during the pandemic found no increased risk of psychological distress (Ervasti et al., 2021). While that study examined restructuring and included public sector employees in stable jobs, we focused on potential for job loss due to downsizing, bankruptcy and notice of layoff. Previous research has found support for a dose-response relationship between the severity of the change (layoffs vs no layoffs), the size of the change and subsequent mental health consequences among the employees (Dahl, 2011; Fløvik et al., 2019). Potentially it is the distinction between our measures that contribute to our diverging findings. It could also be the two different samples with slightly different characteristics.

In our study, those belonging to the group exposed to downsizing represents both survivors of workplace downsizing and employees who avoided dismissal or unemployment through job change. Other studies on survivors of downsizing and layoffs during the COVID-19 pandemic have focused on work-related behaviors, showing decreased commitment and performance (Samreen, Nagi, Naseem, & Gul, 2022; Tu et al., 2021). However, they have not focused on health outcomes. It is possible though that the explanatory factors identified in these studies, including increased job uncertainty, perceptions of fair and transparent leadership and organizational support, could also have served as contributory factors to the elevated risk of anxiety observed in our study. At least these factors corroborate previous knowledge about mechanisms linking organizational downsizings to mental health consequences among survivors of downsizing (Kivimäki, Vahtera, Pentti, & Ferrie, 2000).

When we took into account a person’s prior mental health status, findings on downsizing, furlough and mental health largely remained the same, while the association between job loss/unemployment and mental health attenuated and crossed unity. Although a neglection of prior mental health problems contributes to unclarity about the direction of association, it could also be a type of overadjustment. Some of those prior mental health problems may well have been caused by earlier job instability or by other poor psychosocial working conditions, and are thus part of the main association. However, there is also the possibility that the effect of the economic crisis on mental health is somewhat larger for those without prior mental health problems compared to those with prior psychiatric morbidity. (Pan et al., 2021) In previous, similar studies conducted during the COVID-19 pandemic, baseline or prior mental health problems have been of moderate importance for the main association of job instability and mental health. (Abrams et al., 2022; Ferry et al., 2021; Grandey et al., 2021; Wels et al., 2022) Although a dichotomous outcome of depression is straightforward and more easily interpreted than a continuous measure, changes in the higher and the lower spectrums are not captured if the cut-off point is not crossed in the dichotomized variable. Therefore, we cannot be sure about whether those with prior mental health experienced worse or more symptoms. The stratified analysis did not support effect modification by prior mental health problems though. At the same time, the number of cases with prior mental health problems in each of the respective instability measures were small, likely limiting the statistical power in these analyses.

### 4.1 Strengths and limitations

In the present study we report about possible consequences of job instability on two overlapping but distinct types of mental health problems, depression and anxiety, measured using two validated self-reporting scales, PHQ9 and GAD7. Although anxiety and depression are overlapping and intertwined conditions, it is possible that our results are attributed to some differences between the two. While anxiety oftentimes is associated with worries of the future and in many cases precedes a depression, depression can often follow experienced losses (Pomerantz & Rose, 2014; Wittchen, Kessler, Pfister, & Lieb, 2000). Correspondingly, organizational downsizings often precede a job loss, and can engender worries about the future job situation. A job loss, however by definition implies a loss, both of the work itself and its functions. Although focusing on caseness of depression and anxiety will have masked changes in the lower and upper parts of the scale, the inclusion of both anxiety and depression provides a more nuanced and time sensitive picture on potential mental health consequences from job instability. Although relying on self-reports for both exposure and outcome from the same data source increases the risk of common method bias, it is likely that exposure preceded the outcome in our study. The exposure concerned experiences of furlough, job loss, downsizing etc. at any timepoint during the pandemic, whereas the assessment of mental health problems concerned symptoms during the past two weeks. The ordering of events was likely improved by including information about history of mental health problems, although it might have resulted in overadjusted estimates. It is still uncertain if we captured a change in mental health problems among the exposed. Instead, a logistic regression model with fixed effects, estimating the within-person change in job instability and in mental health while eliminating confounding by time-invariant factors (e.g., previous or chronic morbidity), would have been preferred in order make a more causal interpretation of the results. Unfortunately, our sample did not allow us to properly run such a model as it did not contain enough variation in anxiety nor in depression across our two waves.

The recruitment of participants via an already ongoing panel on work and health likely contributed to a more representative sample than if having relied on snowball sampling or self-enrollments. A limitation though is that respondents with weaker labor market attachments are underrepresented in this cohort which was further reinforced during the pandemic, and which may limit the generalizability of our findings. And although missing data on our sociodemographic factors did not exceed 2%, missing on prior mental health was approximately 15%. We assessed the demographic characteristics of those missing and, as expected, found that men and those with lower socioeconomic status were less likely to respond and attrite from the study. So it may be that we have underestimated the effects of job instability on mental health on the one hand given that lower SES groups are underrepresented, or slightly overestimated the effects given that men less frequently report symptoms of depression. However, sex did not appear to moderate the relationship between job instability and mental health which would indicate underestimations rather than overestimations. In addition, the generalizability also needs to be considered in the light of Sweden's generous social security and job retention schemes, but at the same time fairly liberal standpoint towards public health restrictions during the pandemic, even in relation to the other Nordic Countries (Gordon et al., 2021; Sheridan, Andersen, Hansen, & Johannesen, 2020).

Unfortunately, the sample size was also too small to fully investigate potential effect modifiers and to further refine the job instability continuum. Even though we specifically asked about dismissal, notice of layoff workplace bankruptcy, which ideally could have been included along the continuum, too few respondents reported these experiences and were thus collapsed with the downsizing group. The operationalization of job instability may also suffer from misclassification. Similar to other studies, SLOSH-Corona respondents experienced multiple job instability events, thus their statuses were not necessarily mutually exclusive but characterized by fuzzy boundaries (Burchell et al., 2020; Wang et al., 2022). Furthermore, in the context of the COVID-19 pandemic, no group is completely unexposed to job instability or an unchanged work situation; everyone was affected in one way or another. This may have contributed to reducing the contrast between employees “exposed” and “unexposed” to job instability, potentially underestimating the mental health consequences by these measures of job instability. Lastly, additional residual confounding cannot be ruled out.

## 5 Conclusion

Our findings indicate that some experiences of job instability during the COVID-19 pandemic were associated with mental health problems. However, it was primarily the experience of downsizing and job loss that seemed detrimental to mental health while being furloughed did not seem to have adverse mental health consequences. These findings thus give preliminary support for job retention schemes in the form of short time work allowances as implemented in Sweden during the studied time period, as one possible strategy for job protection, but also to help prevent mental health problems among employees during economic crises.

## Credit author statement

**Sandra Blomqvist**: Conceptualization, Methodology, Data curation, Software, Investigation, Formal analysis, Writing – original draft, Visualization, Project administration. **Robin Högnäs**: Conceptualization, Methodology, Formal analysis, Writing – review & editing. **Marianna Virtanen**: Conceptualization, Validation, Writing – review & editing. **Anthony LaMontagne**: Conceptualization, Validation, Writing – review & editing. **Linda Magnusson Hanson**: Conceptualization, Methodology, Investigation, Resources, Writing – original draft, Validation, Supervision, Project administration, Funding acquisition.

## Ethical statement

The SLOSH Corona study was given ethical permission by the Stockholm County Regional Ethics Committee (Dnr: 2021–04569) and informed consent was obtained from all study participants. SLOSH Corona is an extension to regular the SLOSH data collection which also has received ethical approvals by the Stockholm County Regional Ethics Committee (Dnr: 2012/373-31, 2017/2535-32, 2019–06331, 2022-0156-02).

## Funding

The study was funded by 10.13039/501100002706AFA Insurance, grant number [# 200400].

## Data Availability

The authors do not have permission to share data.
