# Supplementary material for: Job loss and job instability during the COVID-19 pandemic and the risk of depression and anxiety among Swedish employees
Source: SSM Popul Health. 2023 May 4;22:101424. doi: 10.1016/j.ssmph.2023.101424 (PMC10158169; doi:10.1016/j.ssmph.2023.101424)
Supplement: Multimedia component 1 [file mmc1.docx]

| Supplementary Table 1. Comparison of baseline characteristics between individuals lost to follow-up and individuals participating in both waves of data collection. | | | | | |
| --- | --- | --- | --- | --- | --- |
|  | Participating *only* in Wave 1  n=322 | | Participating in Wave 1 *and* Wave 2  n=1580 | | Test of differences (chi-square and t-test) |
|  | N(mean) | %(sd) | N(mean) | %(sd) | p-value |
| Women | 173 | 54 | 883 | 56 | 0.4773 |
| Age | 56 | 12 | 59 | 11 | 0.1802 |
| Married/cohabiting | 83 | 26 | 352 | 23 |  |
|  | 238 | 74 | 1211 | 77 | 0.1964 |
| Compulsory education | 11 | 3 | 54 | 3 |  |
| Upper secondary education | 110 | 34 | 510 | 32 |  |
| University level education | 201 | 63 | 1014 | 64 | 0.8109 |
|  |  |  |  |  |  |
| Household income |  |  |  |  |  |
|  |  |  |  |  |  |
| Manual employee | 63 | 20 | 262 | 17 |  |
| Non-manual employee | 251 | 80 | 1280 | 83 | 0.1916 |
|  |  |  |  |  |  |
| Born outside of Sweden | 18 | 6 | 79 | 5 | 0.8951 |
|  |  |  |  |  |  |
| Pre-pandemic conditions: |  |  |  |  |  |
| Depression | 10 | 4 | 46 | 3 | 0.7021 |
| Anxiety | 40 | 15 | 181 | 13 | 0.3835 |
|  |  |  |  |  |  |
| In work before the pandemic | 241 | 75 | 990 | 63 | **<.0001** |
|  |  |  |  |  |  |
| Mental health at Wave 1: |  |  |  |  |  |
| Scores of depression (PHQ9) | 3.3 | 4.3 | 3.1 | 4.1 | 0.1629 |
| Scores of anxiety (GAD7) | 2.6 | 3.7 | 2.2 | 3.4 | 0.0579 |
